# Define the Two Molecular Subtypes of Epithelioid Malignant Pleural Mesothelioma

**DOI:** 10.3390/cells11182924

**Published:** 2022-09-19

**Authors:** Umair Ali Khan Saddozai, Fengling Wang, Saadullah Khattak, Muhammad Usman Akbar, Muhammad Badar, Nazeer Hussain Khan, Lu Zhang, Wan Zhu, Longxiang Xie, Yongqiang Li, Xinying Ji, Xiangqian Guo

**Affiliations:** 1 Department of Preventive Medicine, Institute of Bioinformatics Center, Henan Provincial Engineering Center for Tumor Molecular Medicine, School of Basic Medical Sciences, Henan University, Kaifeng 475004, China; 2 Gomal Center of Biochemistry and Biotechnology, Gomal University, Dera Ismail Khan 29050, Pakistan; 3 Department of Anesthesia, Stanford University, 300 Pasteur Drive, Stanford, CA 94305, USA

**Keywords:** mesothelioma, gene expression, molecular subtype, subtype-specific treatment

## Abstract

Malignant pleural mesothelioma (MPM) is a fatal disease of respiratory system. Despite the availability of invasive biomarkers with promising results, there are still significant diagnostic and therapeutic challenges in the treatment of MPM. One of three main mesothelioma cell types, epithelioid mesothelioma makes up approximately 70% of all mesothelioma cases. Different observational findings are under process, but the molecular heterogeneity and pathogenesis of epithelioid malignant pleural mesothelioma (eMPM) are still not well understood. Through molecular analysis, expression profiling data were used to determine the possibility and optimal number of eMPM molecular subtypes. Next, clinicopathological characteristics and different molecular pathways of each subtype were analyzed to prospect the clinical applications and advanced mechanisms of eMPM. In this study, we identified two distinct epithelioid malignant pleural mesothelioma subtypes with distinct gene expression patterns. Subtype I eMPMs were involved in steroid hormone biosynthesis, porphyrin and chlorophyll metabolism, and drug metabolism, while subtype II eMPMs were involved in rational metabolism, tyrosine metabolism, and chemical carcinogenesis pathways. Additionally, we identified potential subtype-specific therapeutic targets, including CCNE1, EPHA3, RNF43, ROS1, and RSPO2 for subtype I and CDKN2A and RET for subtype II. Considering the need for potent diagnostic and therapeutic biomarkers for eMPM, we are anticipating that our findings will help both in exploring underlying mechanisms in the development of eMPM and in designing targeted therapy for eMPM.

## 1. Introduction

Malignant pleural mesothelioma (MPM) represents a rare and violent neoplasm that primarily affects the pleural cavity [1,2]. It is a male-dominating disease, with almost 80% of cases occurring due to occupational or environmental exposure to asbestos [3,4,5,6,7]. Genetic susceptibility attached to asbestos exposure has recently been identified as a major factor in the development of malignant mesothelioma. The high number of micronuclei present in the peripheral blood lymphocytes of malignant mesothelioma patients could be a useful index to identify individuals’ susceptibility to the malignancy [8].

MPM has a poor prognosis rate, with a median survival between 6 and 12 months, and less than 5% of the 5-year survival rate [9,10,11]. Different therapies such as first-line therapy (1L), platinum chemotherapy, second-line immunotherapy (2L), or maintenance therapy are applied in clinical trials, but their outcomes are not promising [12,13,14]. Furthermore, only a small number of patients can be cured through surgery due to the late diagnosis of the disease [15,16]. Recently, significant studies on the carcinogenic behavior of asbestos and other fibers, and the genetic background of MPM have led to better understanding of the disease [17,18,19,20,21,22,23]. According to the 2015 World Health Organization (WHO) histological lung and pleura tumor classification, malignant mesothelioma is classified into three major histological subtypes namely epithelial, biphasic and sarcomatoid [24,25], with the largest proportion of epithelioid mesothelioma that has a better outcome as compared to the sarcomatoid and mixed type. Based on the response to treatment, epithelioid mesothelioma is heterogeneous. To promote the efficiency of recent therapies, finding ways to profile this group of patients with more accuracy, is vital for personalized treatment and new therapeutic options [26]. Different practices are under development to guide the treatment of cancers. For example, the recently developed gene expression profiling methods are used to facilitate the diagnosis and management of breast cancer, gastric cancer, leiomyosarcoma and pheochromocytoma [27,28,29,30,31,32,33]. The effective classification of cancers into distinct molecular subtypes helps cancer patients to have an improved diagnosis and to obtain more effective remedies [34]. So far, limited data about eMPM are enough for comparing it with different molecular subtypes. The current study used gene expression profiling data for molecular subtyping eMPM, two common eMPM molecular subtypes were measured, defined, and solidified with the 39 cases of the GSE29354 dataset and 57 cases of the TCGA dataset. Further identified various therapeutic genes and pathways in the analyzed molecular subtypes that may help develop the new target therapy specific to the eMPM molecular subtype.

## 2. Materials and Methods

### 2.1. Determination and Validation of Molecular Subtypes of eMPM

The expression profile of clinical eMPM cases was derived from TCGA and Gene Expression Omnibus (GEO) databases. The molecular subtypes of eMPM were defined based on two datasets, one from TCGA (57 cases) and the other of (GSE29354) from GEO (39 samples). After the individual expression datasets were filtered with standard deviation, gene-based centring was performed to transform the data. Using the Consensus clustering package Consensus Clustering Plus, both datasets were run separately through Consensus clustering with the following parameters: 80% resampling of the samples, 80% resampling of the genes, a maximum evaluated k of 12 after genetic clustering, distance (1-Pearson correlation), the agglomerative hierarchical clustering algorithm, and 1000 iterations to identify molecular subtypes [35]. Lastly, the accuracy of subtype assignment from Consensus Clustering Plus was determined by R package cluster (silhouette width) [36].

### 2.2. Reproducibility Measurement of eMPM Molecular Subtypes

The reproducibility of eMPM molecular subtypes between TCGA and GSE29354 cohorts was determined through Subclass Mapping (SubMap) implemented in GenePat-tern, with parameters of Num.marker.Genes = 300, num.perm = 1000 and num.per.fisher = 1000 [37].

### 2.3. Gene Ontology and Gene Set Enrichment (Gsea) Analysis

SAM [38] and SAMseq [39], with less than 0.05 false discovery rate, were applied to identify genes specific to each subtype. DAVID Bioinformatics Resources Online version 6.7 (https://david.ncifcrf.gov/ (accessed on 1 July 2022)) (accessed on 1 July 2022)) was also used for GO and KEGG pathway analysis. GSEA [40] analysis was used to examine the expression levels of genes and pathways particular to every subtype. Moreover, the TARGET V2 database (http://www.broadinstitute.org/cancer/cga/target (accessed on 1 July 2022)) explored the potential therapeutic genes of each eMPM subtype.

### 2.4. Statistical Analysis

To evaluate whether the association between clinical factors and subtypes of eMPM is statistically significant, Fisher exact test and chi-square test were applied, and a *p*-value less than 0.05 was considered significant. In addition, the Kaplan–Meier plot and log-rank test were performed by Graphpad Prism 7 software. The seven targeted genes were also analyzed by GEPIA to assess the overall survival (OS) and prognostic value of these genes [41].

## 3. Results

### 3.1. Consensus Clustering Identified Two Different Empm Molecular Subtypes

**Figure 1:**
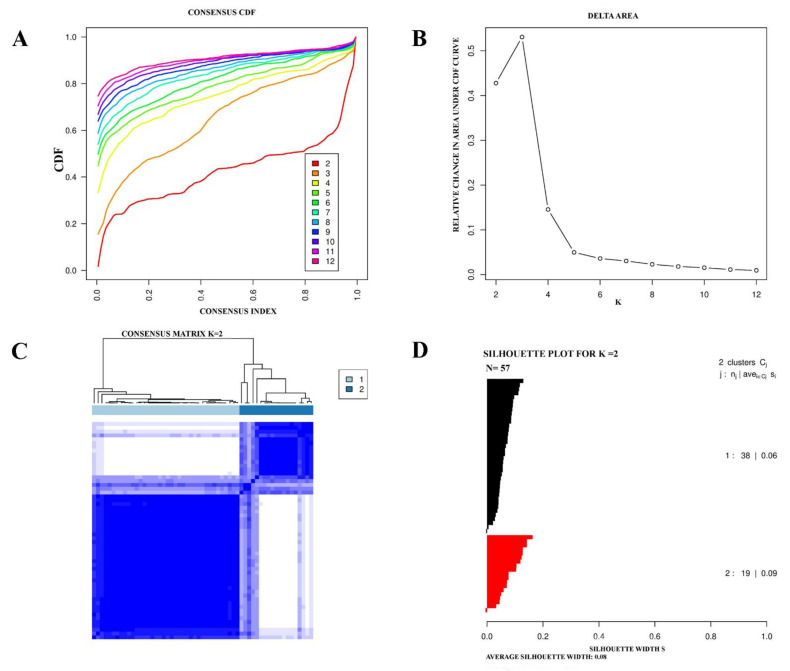
The TCGA cohort of eMPM, represented two molecular subtypes. (**A**) Using an empirical cumulative distribution plot, the optimal number of eMPM molecular subtypes was found. (**B**) Comparative increase in the area under the CDF curve along with increasing expected number of molecular subtypes. (**C**) Matrix of Consensus clustering for the two unique eMPM subtypes. (**D**) Silhouette plot of eMPM based on Consensus clustering assignment.

Consensus clustering was used for the identification of eMPM subtypes based on their gene expression profiles. Initially, the curve of empirical cumulative distribution (CDF) (Figure 1A–C) revealed that the TCGA cohort with 57 eMPM samples has two optimal molecular subtypes. The results of Consensus clustering were confirmed by silhouette width analysis. It was found that 55 out of 57 samples had a positive silhouette value, thus used for later analysis. Among 55 samples, 44 were of subtype I, while 11 samples belonged to subtype II (Figure 1D).

### 3.2. Validation of eMPM Molecular Subtypes by Independent Dataset

**Figure 2:**
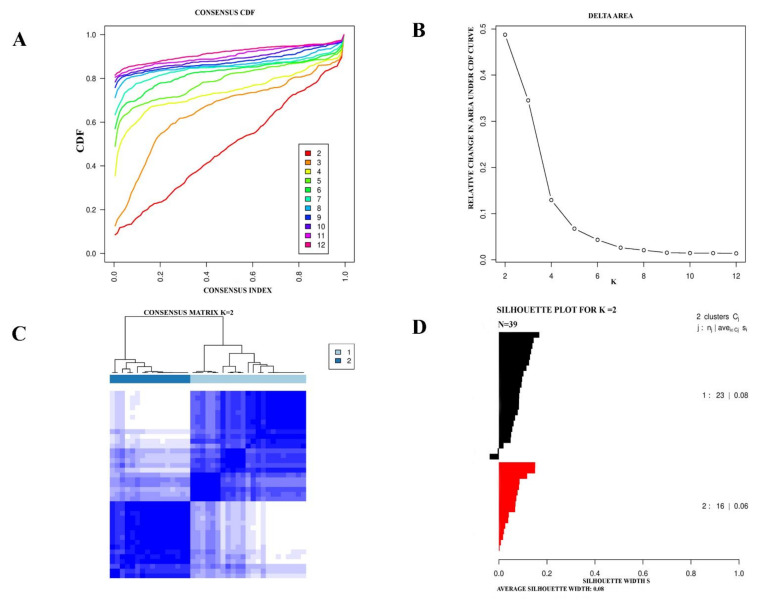
The TCGA cohort of eMPM represented two molecular subtypes. (**A**) An empirical cumulative distribution plot used to determine the optimal number of molecular subtypes for eMPM (**B**). Compared to the prediction of the number of molecular subtypes found using the CDF curve, the area under the CDF curve has increased (**C**). For the two distinct eMPM subtypes, a Consensus clustering matrix has been developed (**D**). Based on the Consensus grouping assignment, a silhouette plot of the eMPM has been created.

The two subtypes of eMPM from the TCGA cohort were further verified by analyzing a GEO dataset (GSE29354) with 39 eMPM cases. In the GSE29354 dataset, Consensus clustering did also identify two molecular subtypes (Figure 2). Like the TCGA dataset, positive silhouette cases obtained in the GSE29354 dataset were used for further analysis.

### 3.3. SubMap Analysis of Molecular Subtypes in Independent Empm Cohorts

**Figure 3:**
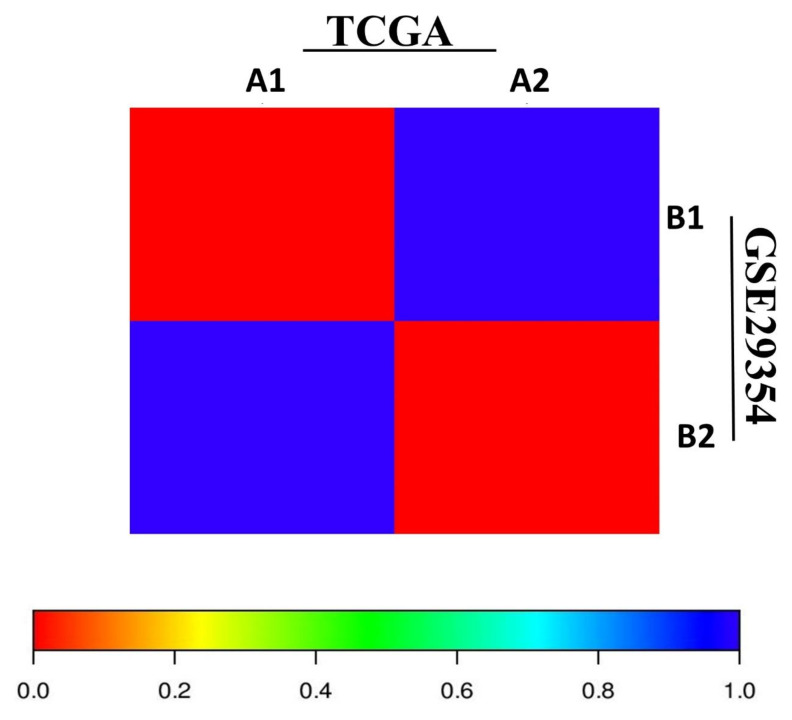
A significant association between the two molecular subtypes of eMPM from the two independent datasets of TCGA and GSE29354. The correlation significance was expressed as an FDR-corrected *p*-value.

SubMap analysis was performed to evaluate the correlation between two different molecular eMPM subtypes in independent datasets. It was revealed that the A1–A2 subtypes of TCGA were significantly correlated with the B1–B2 subtypes of GSE29354 (Figure 3). This indicates that the molecular subtypes are common and reproducible across different eMPM cohorts.

### 3.4. Clinical Characteristics of eMPM Molecular Subtypes

The eMPM molecular subtypes in the TCGA cohort were clinically characterized by studying the relationship between these subtypes and their clinical factors. It was found that right sides had a remarkably higher laterality rate for both eMPM subtypes, which is 72.2% in subtype I and 54.5% in subtype II (Supplementary Table S1, *p* = 0.3492). In this dataset of eMPM the number of patients from subtype I was found to be higher (44/57) as compared to the subtype II (11/57) (*p* = 1) and a maximum number of patients belongs to stage III cancer in which subtype I found at high risk that is (54.5%) (*p* = 0.58).

**Figure 4:**
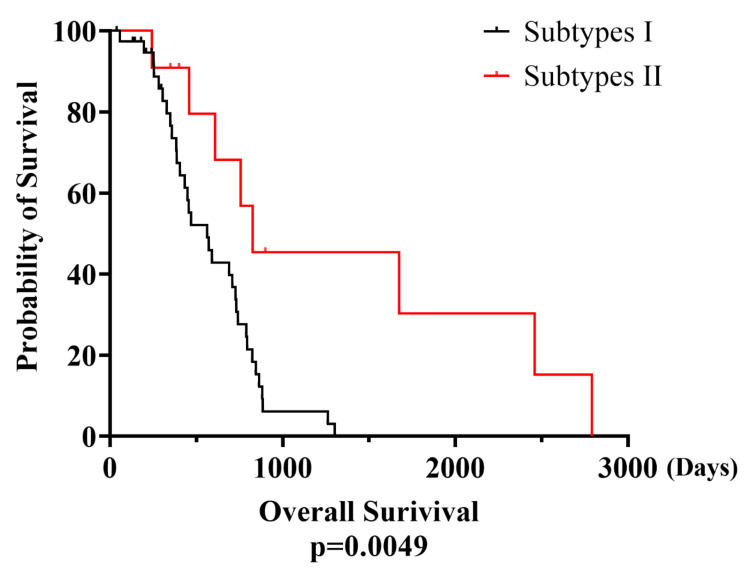
Kaplan–Meier plot for survival time of subtype I patients (Red) and subtype II patients (black).

**Figure 5:**
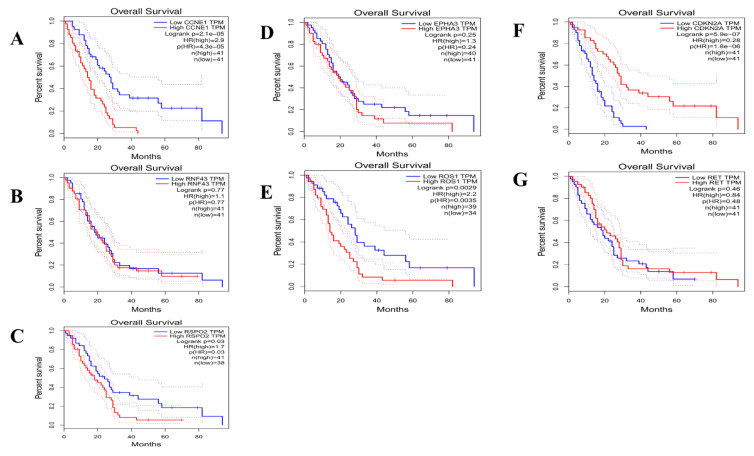
Kaplan–Meier curves of targeted genes expression based on TCGA in GEPIA. Blue curve shows low expression, and red curve show high expression. Whereas (**A**–**E**) targeted genes belong to Subtype I and (**F**,**G**) belong to Subtype II of Epithelioid malignant pleural mesothelioma. Logrank *p* < 0.05 was considered to be significant.

Whereas 463 days were recorded as the median overall survival time (OS) of eMPM subtype II patients, the time slightly shorter than the survival time of subtype I patients, which was recorded to be of 791 days (*p* = 0.0049*). Kaplan–Meier plots (KMP) curve analysis showed significant difference between the overall survival rates of the two eMPM subtypes. The red line of KMP represents the OS of subtype II patients while the black line represents the OS of subtype I patients (Figure 4). (Supplementary Table S1). We further found that all the targeted genes with low and higher expression levels presented different overall survival (OS) in both subtypes of epitheloid malignant pleural mesothelioma. Higher overexpression of all 5-subtype I including *CCNE1, EPHA3, RNF43, ROS1* and *RSPO2* genes show low overall time survival and lower expression levels show the longer overall survival time. Whereas the higher rate of overexpression in two target genes (*CDKN2A* and *RET*) represent high OS and lower expression show the small OS time in mesothelioma. The significance rate of *CCNE1* (*p* = 2.1 × 10^−5^*)*, EPHA3* (*p* = 0.25)*, RNF43* (*p* = 0.77)*, ROS1* (*p* = 0.0035*) and *RSPO2* (*p* = 0.03*) for subtype I and *CDKN2A* (*p* = 5.9 × 10^−7^*) and *RET* (*p* = 0.46) for subtype II (Figure 5).

### 3.5. Functional Analysis of eMPM Subtype-Specific Genes

Differential gene expression of two eMPM molecular subtypes in the TCGA dataset was analyzed by SAMseq analysis. It was revealed that 1520 genes had differential expression between the two subtypes, out of which 1161 genes had overexpression in subtype I and down-expression in subtype II eMPM. In contrast, the 359 genes were upregulated in subtype II and down-regulated in subtype I (Supplementary Table S2).

**Figure 6:**
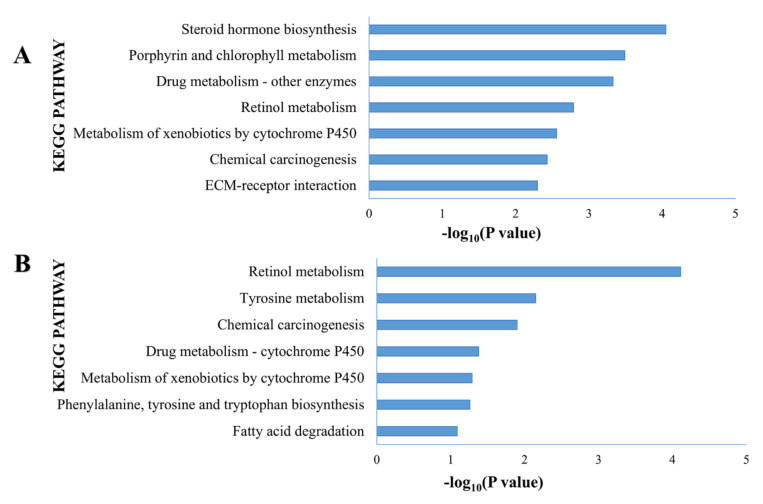
Pathways enriched in epithelioid malignant plural mesothelioma subtypes. (**A**) Subtype I KEGG pathways of eMPM. (**B**) KEGG pathways in subtype II eMPM.

**Figure 7:**
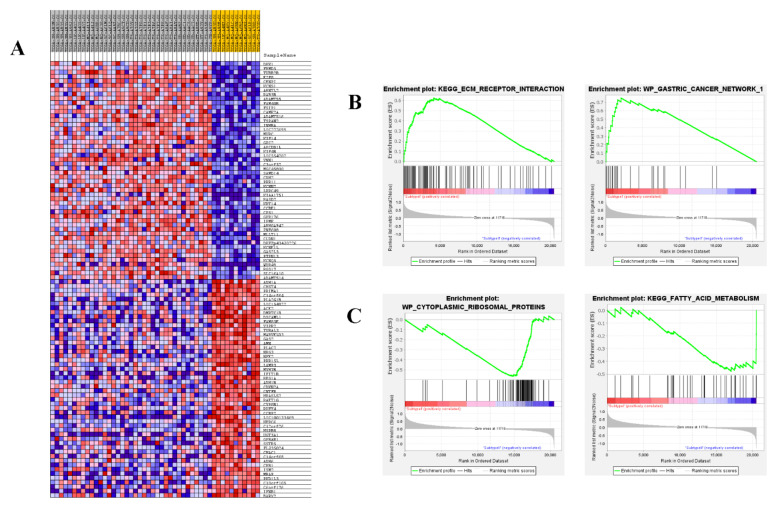
Different gene expression signatures in different eMPM molecular subtypes are shown by GSEA. (**A**) Different gene expression patterns in subtype I and subtype II are represented. Genes that are overexpressed are red, while genes that are under-expressed are blue. (**B**) In subtype I, GSEA demonstrates the activity of the ECM receptor interaction and WP gastric cancer network. (**C**) In subtype II, GSEA revealed the activation of the fatty acid metabolism pathway and cytoplasmic ribosomal proteins pathways.

The top 200 upregulated genes from each eMPM subtype were further analyzed by KEGG and GO to find additional biological information about the subtypes. Subtype I was revealed by GO analysis to be enriched with 50 different biological processes, including negative regulation of glucuronosyltransferase activity (2.73%) (Supplementary Table S3). Analysis of upregulated genes in subtype I by KEGG revealed 15 different pathways belonging to steroid hormone biosynthesis, porphyrin and chlorophyll metabolism, drug metabolism, and other enzyme pathways, etc. (Figure 6A). In the case of subtype II eMPM, it was found to be significantly enriched with 32 biological processes and 7 KEGG pathways, including rational metabolism, tyrosine metabolism, chemical carcinogenesis pathways, etc. (Figure 6B). Moreover, demonstration of TCGA cohort gene sets by GSEA analysis showed that 2922 gene sets with GSEA in TCGA dataset, 1979 gene sets were shown to be enriched in two subtypes, with 1568 of them overexpressed in subtype I and the remaining 411 overexpressed in subtype II (Table 1). Besides this, subtype I was rich in significant biological pathways, including ECM receptor interaction and WP gastric cancer network. Subtype II analysis found pathways of fatty acid metabolism and pathway of cytoplasmic ribosomal proteins (Figure 7B, C).

### 3.6. Clinical Implication of eMPM Subtyping

The molecular subtyping of eMPM was performed to search and identify therapeutic ways and to apply these specified routes for further clinical studies and discourses. For the determination of therapeutic molecules, the genes upregulated in both eMPM subtypes were compared with the TARGET database containing target genes and functional inhibitors [11]. However, further studies may be required for the targeted genes to be translated into potential clinical stages [42,43,44]. The current study has found seven target genes specific to each subtype, based on which eMPM patients from certain subtypes would gain relative benefits. A total of five target genes, namely *CCNE1, EPHA3, RNF43, ROS1,* and *RSPO2,* can benefit subtype I eMPM patients, while subtype II patients can benefit from two target genes, i.e., *CDKN2A* and *RET* (Table 2).

## 4. Discussion

The most common and primary type of pleural malignancy is malignant pleural mesothelioma (MPM). It exhibits a poor prognosis because of its highly aggressive clinical nature [45]. Early diagnosis of MPM may increase the survival rate of MPM patients. Presently, it is hypothesized about the treatment of MPM in different studies that even the administration of identical treatment to the patients at the same stage of the disease may result in different responses owing to molecular heterogeneity [46,47].

Molecular subtyping of tumors based on gene expression profiling has aided in the development of subtype-specific diagnosis, prognosis, and therapies [35]. A suitable example of subtype targeted therapies is the herceptin treatment trial in breast cancer. Patients with HER2-negative breast cancer did not benefit from herceptin treatment, whereas those with HER2-positive breast cancer responded substantially and benefited from it [48].

Though the World Health Organization (WHO) classifies MPM into epithelioid, sarcomatoid, and mixed (biphasic) subtypes, the actual spectrum of tumors is completely overgeneralized by this division. Although the epitheloid subtype possesses a limited number of prognosis and survival data, this study took a better step to use the limited data and identify the molecular subtypes based on gene expression profiles, and propose proper targeted therapies for eMPM.

The gene expression profiling method can make it possible to characterize the biological diversity of eMPM, and it also provides the opportunity for the development of therapeutic strategies specific to the subtype.

This study identified two molecular eMPM subtypes [49](also confirmed by de Reyniès et al.). The gene expression profiling method revealed 39 cases in the GSE29354 dataset and then validated in the TCGA cohort with 57 eMPM cases. In both of these, certain specific genes and pathways were revealed by gene set enrichment and gene ontology analyses to be overexpressed (Supplementary Table S4). Genes overexpressed in subtype I eMPM included *DKK1* and *CPS1* (Figure 7), enriched the pathways including steroid hormone biosynthesis, porphyrin, chlorophyll metabolism, drug metabolism, etc. (Figure 6). A recent study revealed that the pattern of miRNA expression in MPM is highly uncontrolled, and a 2-miRNA signature might be a potentially helpful tool for MPM prognosis [50]. Data indicated T-type Ca^2+^ channel expression in malignant mesothelioma (Mme) tissue and their participation in epigallocathecin-3-gallate (EGCG)-specific cytotoxicity to MMe cells, implying that these channels might be used as a novel MMe pharmaceutical target [51]. An unanticipated link between ERb-mediated tumor suppression and energy metabolism is another option to improve the treatment of malignant mesothelioma [52]. Owing to its role in regulating tumor progression by inhibiting the classical Wnt pathway [53,54], most studies define *Dkk1* as a biological marker with the potential to evaluate tumor diagnosis and prognosis [55,56,57]. It has been recognized by some studies that *Dkk1* can be overexpressed in several different cancer cell lines, including liver, lung, breast, glioma, and cervical cancer, and it inhibits cell proliferation and differentiation by inducing apoptosis [58,59]. *CPS1* (carbamoyl phosphate synthetase 1) is a multidomain enzymatic protein found in mitochondria, liver, and intestine that catalyzes the first committed step of the urea cycle for ammonia detoxification and disposal [60]. A potent study showed the overexpression of *CPS1* has been linked to both unfavorable therapeutic responses in colorectal cancer patients receiving neoadjuvant concurrent chemoradiotherapy, according to new research (CCRT) [61]. Pham-Danis et al. recently discovered that inhibiting CPS1 with EGFR inhibitors can lower the proliferation of EGFR-mutant non-small cell lung cancer (NSCLC) cells and stop them from progressing through the cell cycle [62]. In addition, The Cancer Genome Atlas (TCGA) has revealed the high expression of the *CPS1* gene in a variety of cancer types, including bladder, colon, esophageal, endometrial, lung, and prostate cancers [63].

Rational metabolism, metabolism of tyrosine, chemical carcinogenesis, etc., are the pathways that are enriched in subtype II, while *LAMP3* is the gene that is overexpressed in this subtype (Figure 6). Primarily, *LAMP3* was reported in lung tissues but is found to have overexpression in multiple primary cancers, including breast, lung, and liver cancers [64]. Moreover, *LAMP3* is considered a suitable biomarker for breast cancer as it is associated with the progress regulating hypoxia [65], and its expression in epithelial cells is reported to evaluate the prognosis of esophageal squamous cell carcinoma [66]. It was also found that *LAMP3* is one of the genes that are highly upregulated in osteosarcoma lung metastasis tissue than in conventional osteosarcoma tissue [67]. Studying the expression of each subtype’s specific genes and pathways will be a better way to understand eMPM at the subtype level and help develop treatment strategies against specific subtypes.

A recent study suggests that disease occurrence takes place primarily from the loss of tumor suppressor gene function, and there are no “druggable” driver oncogenes associated with MPM [68]. However, in our molecular subtyping, different genes and pathways expressed in each subtype were further compared with the TARGET database. For the two eMPM subtypes, seven known target genes were identified (Table 2). Genes, namely CCNE1, EPHA3, RNF43, ROS1, and RSPO2, were found to be overexpressed in subtype I eMPM, whereas CDKN2A and RET were the genes overexpressed in subtype II. Therefore, the subtype-specific molecular characterization might translate into “druggability” in the future.

*CCNE1*, along with its catalytic subunit CDK2, has a key role in regulating the cell cycle, for it assures precise control of DNA replication, chromosome segregation, and the G1 to S-phase transition [69,70]. Expression of *CCNE1* has been reported in various cancers such as bladder cancer [71], colorectal cancer [72], gastric [73], high-grade serous ovarian carcinomas (HGSCs) [74] and ovarian cancer [75]. Due to its relative specificity for cyclin E and the critical function CDK2 plays in the activated CDK2/cyclin E1 complex, CDK2 is an appealing target in the treatment of *CCNE1* amplified malignancies. In vitro, the targeted inhibition of CDKs using pan-CDK inhibitors and, more specifically, CDK2 inhibitors have shown promise in CCNE1-amplified malignancies [76,77]. Pan-CDK inhibitor, e.g., dinaciclib (SCH-727965), has been clinically tested to inhibit CDKs 2/5/1/9 involved in hematological and solid malignancies (NCT00798213 and NCT00937937) [78,79]. A similar outcome was also reported with the correspondence to the survival rates and detection of cancer. There are several oncogenic proteins such as *EPHA3* and *RSPO1, RSPO2,* and *RSPO3,* which are overexpressed in lung adenocarcinomas and lymphoblastic leukemia and define the patient’s survival rates [80,81,82]. Similarly, mutations of *RNF43* and *RNF43/ZNRF3* or *RSPOs* also play a pivotal role in the activation of oncogenic pathways in various cancers and determine the cancer onset [83,84,85,86,87]. Hence, multiple approaches are being developed by using these marker proteins as therapeutic targets. For example, dasatinib is used in the activation of EphA3 via mediating role of the ABL1 protein kinase domain in lymphoblastic leukemia, and PORCN is considered suitable for the palmitoleation of mammalian Wnts to treat ovarian cancer. These targets are being extensively explored for better understanding and are evaluated in clinical trials in oncological settings [88,89,90,91,92,93,94,95]. Another latest study of malignant pleural mesothelioma (MPM) also presented the CD74-ROS1 fusion for the first time with a complete and long-term response to crizotinib [96]. Thus, the possible use of these inhibitors may play a significant role in subtype I of eMPM.

P16INK4a encoded by cyclin-dependent kinase (CDK) inhibitor 2A (CDKN2A) regulates the cell cycle by inhibiting CDK4/6. Mutation or loss of *CDKN2A* has been detected in various malignancies and results in increased CDK activity [97]. *CDKN2A* deletion frequency in MPM varies from 61% to 88% in primary tumors, with some studies suggesting deletion in only one-fifth of the cases of MPM [98,99,100,101,102,103,104]. The arrival of FDA-approved anti-CDK4/6 inhibitors (palbociclib and ribociclib) for breast cancer metastasis has made it targeting cell cycle dysregulation more accessible [105]. Palbociclib has also exhibited encouraging results against patients with liposarcoma [106] and *CDKN2A*-mutated non-small cell lung cancer [107]. Moreover, tyrosine kinase receptor (TKR)-targeted antibodies and tyrosine kinase inhibitor (TKI) related micro molecules have aroused another ray of hope in treating different cancers by blocking the TKR and subsequent molecular signaling [108]. The rearranged during transfection (RET) proto-oncogene can act as a potential therapeutic TKR target for TKIs such as carbozantinib, vandetanib, sunitinib, and sorafenib. They are primarily targeted in thyroid carcinomas and NSCLC’s RET. Patients suffering from malignant mesothelioma (MM) can also benefit from similar therapeutic targeting [109].

To the best of our knowledge, the current study revealed the overexpression of *CDKN2A* first time in eMPM. Therefore, investigating the role of these inhibitors in eMPM and patient selection based on such molecular characteristics becomes vital for the success of future clinical studies.

In conclusion, we defined distinct intrinsic molecular subtypes of eMPM with different gene signatures in two independent cohorts. Our finding provides insight into the understanding of the malignancy development and progression of eMPM and provides valuable information to develop individualized subtype-specific therapies for eMPM.

## Figures and Tables

**Table 1:** Number of overexpressed and down-expressed genes set in eMPM.

	Subtype I	Subtype II
**1979 gene sets**	1568	411
**FDR < 25%**	175	68
**Nominal *p*-value < 5%**	245	81
**Nominal *p*-value < 1%**	141	47

**Table 2:** Target genes enriched in each molecular subtype of eMPM.

Gene Overexpressed		Examples of Potential Therapeutic Agents
	***CCNE1***	CDK2 inhibitor
	*EPHA3*	Dasatinib, Ephrin inhibitors
**Subtype I**	*RNF43*	Porcupine inhibitors
	*ROS1*	Crizotinib
	*RSPO2*	WNT inhibitors
**Subtype II**	*CDKN2A*	CDK4/6 inhibitors
	*RET*	Sorafenib, vandetinib, RET inhibitors

## Data Availability

The datasets presented in this study can be found in online repositories. The names of the repository/repositories and accession number(s) can be found in the article/Supplementary Material.

## Supplementary Materials

The following supporting information can be downloaded at: https://www.mdpi.com/article/10.3390/cells11182924/s1, Table S1: Clinicopathologic Characteristics of eMPM (N = 57). Table S2: Results of SAM analysis between different subtypes of eMPM in TCGA. Table S3: Biological process enriched in each subtypes of Mesothelioma. Table S4: Biological process (KEGG) enriched in each subtypes of Mesothelioma.
