# Supplementary material for: Define the Two Molecular Subtypes of Epithelioid Malignant Pleural Mesothelioma
Source: Cells. 2022 Sep 19;11(18):2924. doi: 10.3390/cells11182924 (PMC9497219; doi:10.3390/cells11182924)
Supplement: Supplementary file 1 [file cells-11-02924-s001.zip › cells-1854477-Table S1.pdf]

Table S1. Clinicopathologic Characteristics of eMPM (N=57)

**Characteristic**	**Patients, n(%)**	**Subtype I**	**Subtype II**	**Others**	***P value***
**Total number**	57	44	11	2	
**Age (year)**					
Mean	61.08	60.09	64.45	64.5	0.365
Range	28-81	28-81	54-75	60-69	
**Sex**					
Female	13	11	2	0	1
Male	44	33	9	2	
**Laterality**					
Left	22	17	3	2	0.3492
Right	32	24	8	0	
Bilateral	3	3	0	0	
**Disease detection**					
Biopsy	34	26	7	1	0.2861
Cytology	2	1	0	1	
Thoracentesis	1	1	0	0	
Thorascopy	9	6	3	0	
**Stages**					
I	5	4	1	0	0.588
IA	1	1	0	0	
II	12	8	4	0	
III	29	24	3	2	
IV	10	7	3	0	
**Radiation therapy**					
Yes	19	15	3	1	0.878
No	37	28	8	1	
**Days to death**					
Median	567.5	463	791.5	64.5	0.0049*
Range	39-2790	39-1302	244-2790	361-949	
**Event**					
Alive	11	8	3	0	0.788
Dead	46	36	8	2	
