# Supplementary material for: Define the Two Molecular Subtypes of Epithelioid Malignant Pleural Mesothelioma
Source: Cells. 2022 Sep 19;11(18):2924. doi: 10.3390/cells11182924 (PMC9497219; doi:10.3390/cells11182924)
Supplement: Supplementary file 1 [file cells-11-02924-s001.zip › cells-1854477-Table S2.pdf]

Table S2. Results of SAM analysis between different subtypes of eMPM in TCGA.
Data is representing overexpressionof gene in two subtype in positive and minus value.
(1).Gene were ordered according the SAM significance, gene with the positive value were
overexpressed in subtype II of eMPM whereas minus value genes were overexpressed in subtype I.
(2).Significance increased with decreased of absolute values of ranks from 10 to 1 or -10-1.Rank 1 or -1 genes are the most significant of SAM results.

Gene symbol	TCGA sItosII	Gene symbol	TCGA sItosII	Gene symbol	TCGA sItosII	Gene symbol	TCGA sItosII	Gene symbol	TCGA sItosII
TLX3	-1161	SLC2A3	-858	TNNI3	-554	LOC283404	-250	KRT39	55
GIPR	-1160	RIPPLY2	-857	CHMP4C	-553	HOXA11	-249	CCBP2	56
DUOX2	-1159	SLC17A8	-856	AMHR2	-552	PCDH10	-248	DMBT1	57
CABP4	-1158	C2orf27A	-855	RTN4RL2	-551	CORO6	-247	FAM19A4	58
C17orf64	-1157	ZBED2	-854	CREB3L1	-550	PRSS35	-246	SLC10A1	59
STK33	-1156	DLX2	-853	ADAMTS4	-549	PTH2R	-245	HS3ST6	60
CDH7	-1155	LOC344967	-852	PURG	-548	PAQR9	-244	KCNJ13	61
CDH18	-1154	FAT4	-851	C11orf70	-547	GSG1	-243	SLC30A2	62
ANKRD34A	-1153	SLC39A14	-850	TNFRSF19	-546	E2F7	-242	CRHR1	63
SLC4A8	-1152	ITGA7	-849	SPATA8	-545	SP7	-241	C6orf176	64
TCN1	-1151	IGLON5	-848	DPYSL4	-544	PPP4R1L	-240	BTC	65
DACT3	-1150	SMC1B	-847	OPN4	-543	LOC100190938	-239	ZNF385B	66
TMEM139	-1149	HMP19	-846	F12	-542	NDRG4	-238	SHISA3	67
B3GNT3	-1148	GPR173	-845	DLGAP5	-541	PRKG1	-237	RBP5	68
SYT14	-1147	REM1	-844	FXYD7	-540	ALDH1A3	-236	FRMPD4	69
PRSS8	-1146	KLF17	-843	TRPS1	-539	PDLIM3	-235	CYP24A1	70
PCDH19	-1145	FOXB1	-842	SLFN13	-538	FSD1	-234	AIPL1	71
ZNF582	-1144	EPOR	-841	LHX2	-537	DPYSL3	-233	HRASLS2	72
UTF1	-1143	CNTN5	-840	RHOBTB1	-536	SGCG	-232	CYP3A4	73
NUAK1	-1142	ESPL1	-839	COL7A1	-535	OVCH2	-231	MAB21L2	74
DUSP7	-1141	ARID5B	-838	IL11	-534	DRP2	-230	NRG4	75
C1QTNF2	-1140	SPRED3	-837	NAV1	-533	ST6GALNAC5	-229	DKFZp779M06	76
SERINC2	-1139	DCBLD1	-836	GREB1L	-532	ZSCAN12P1	-228	DHODH	77
KRT33B	-1138	CFHR3	-835	DAD1L	-531	DNAJC12	-227	SLC24A3	78
SLC19A2	-1137	HTR1F	-834	CREB5	-530	FAM133A	-226	CNTFR	79
ZIC4	-1136	HOXA4	-833	ITGB3	-529	ITGA11	-225	LOC400759	80
COL12A1	-1135	CTH	-832	CNKSR3	-528	KIF14	-224	LPPR1	81
C9orf84	-1134	RSPH4A	-831	UBE2Q2P1	-527	COMP	-223	KCND3	82
PTCH2	-1133	USH1C	-830	FAM19A3	-526	ETV4	-222	CLRN3	83
LOC644538	-1132	NAALADL1	-829	ENAH	-525	PEAR1	-221	SERPINB10	84
FAM101B	-1131	KIF25	-828	CLDN18	-524	KCNT2	-220	ALDH1L1	85
ANGPT1	-1130	RHEBL1	-827	MPP2	-523	BICD1	-219	HSD17B6	86
OSR2	-1129	TDRD9	-826	CLEC5A	-522	LOC283731	-218	RASGEF1C	87
KIF11	-1128	PKDCC	-825	PODNL1	-521	NXPH2	-217	DPYS	88
LOC285847	-1127	OLIG3	-824	CPA5	-520	KIF18B	-216	CHAC1	89
WISP1	-1126	CPEB1	-823	RBMXL2	-519	SLITRK5	-215	NCRNA00113	90
TP63	-1125	CLEC2A	-822	TROAP	-518	QPRT	-214	CEACAM6	91
ANKRD33	-1124	ALDH1L2	-821	GFRA3	-517	NPW	-213	TCEAL2	92
GABRB2	-1123	ITGA2	-820	MMP16	-516	NOX5	-212	HIST1H3H	93
FAM163B	-1122	CTHRC1	-819	TMEM52	-515	KCTD16	-211	LOC441177	94
FABP6	-1121	HAPLN1	-818	C1orf135	-514	CLDN3	-210	SLC5A7	95
LOC100302401	-1120	SLC38A1	-817	TWIST1	-513	BCL2L10	-209	PIP5K1B	96
WDFY2	-1119	NLGN2	-816	AMZ1	-512	HS3ST5	-208	SULT1A2	97
FLJ22536	-1118	LGI1	-815	RALGPS2	-511	CDK5R2	-207	RET	98
MYO16	-1117	ITPRIPL1	-814	KIF5A	-510	C2orf39	-206	ALB	99
FAM55C	-1116	POSTN	-813	LOC283392	-509	KLRG2	-205	SPACA3	100
CHST11	-1115	BUB1B	-812	GDF6	-508	DCDC2	-204	RNF182	101
CASC5	-1114	ADAMTS18	-811	C9orf110	-507	SOST	-203	LAMP3	102
ASTL	-1113	CRP	-810	C1orf110	-506	C12orf53	-202	DRD5	103
SACS	-1112	TERT	-809	C6orf222	-505	CAMKV	-201	WNT7B	104
BARD1	-1111	DSC3	-808	SPC24	-504	EPGN	-200	MYH7B	105
FAM92A1	-1110	RAB9B	-807	POU3F1	-503	CDH17	-199	SSTR5	106
CETN4P	-1109	KLF14	-806	LYPD6B	-502	FZD9	-198	FAM5B	107
MGAT3	-1108	WNT16	-805	A4GNT	-501	UGT1A8	-197	IFIT1B	108
CDC25A	-1107	TMEM40	-804	UBE2C	-500	C9orf70	-196	ADAMTS8	109
SNHG4	-1106	NTM	-803	ELOVL4	-499	FGFBP1	-195	PTPRT	110
SGCD	-1105	GLDC	-802	C15orf26	-498	A1BG	-194	HSH2D	111
JPH2	-1104	MAP1A	-801	SLIT2	-497	GRIA4	-193	SPINK2	112
ILDR2	-1103	GGT8P	-800	RUNX1T1	-496	CNN1	-192	NPY1R	113
TAS2R5	-1102	SLC38A3	-799	FTCD	-495	SAMD14	-191	SERPINI1	114
PI15	-1101	CPLX3	-798	CCDC144C	-494	ACTBL2	-190	TUBA3C	115
CLCN4	-1100	CLCF1	-797	ARHGAP42	-493	TNFRSF9	-189	SLC7A4	116
SUCNR1	-1099	CCDC36	-796	MCF2L2	-492	MAP7D2	-188	CFC1B	117
RELL2	-1098	PCNXL2	-795	LOC647859	-491	GCNT3	-187	RAET1E	118
MRAS	-1097	KLK4	-794	TMEM133	-490	FHAD1	-186	SYT4	119
SNORA59B	-1096	DENND2C	-793	SRPK3	-489	ACAN	-185	COL9A3	120
MMRN1	-1095	CHRM4	-792	GTSE1	-488	KCNE1L	-184	ARMS2	121
GK3P	-1094	CRABP2	-791	NPR3	-487	C11orf88	-183	NTNG1	122
PRR7	-1093	FOXL1	-790	KIF26B	-486	C12orf70	-182	HIST1H2BD	123
CPA4	-1092	C12orf77	-789	S100A2	-485	DGKI	-181	LOC285696	124
CDX1	-1091	IQGAP3	-788	CRTAC1	-484	SLC9A4	-180	POM121L2	125
GP5	-1090	CDCA8	-787	CCDC19	-483	TTK	-179	WSCD1	126
ZDHHC8P1	-1089	C1QL4	-786	SLC6A19	-482	LOC100130933	-178	IRX6	127
PLAT	-1088	C15orf42	-785	SLC39A4	-481	C20orf195	-177	MARVELD3	128
LOC728723	-1087	C2CD4D	-784	SLC13A5	-480	BCAN	-176	TGM1	129
IRF6	-1086	RAB33A	-783	TMEM169	-479	GAS2L3	-175	ITGAD	130
FAM72D	-1085	PRKCG	-782	FAM189A1	-478	GUCY1A2	-174	HIST1H4H	131
COX6B2	-1084	AURKB	-781	SYT11	-477	SCG5	-173	SLC39A8	132
CAMK2N2	-1083	MYLPF	-780	HAPLN4	-476	NPTX2	-172	PHACTR3	133
NODAL	-1082	PELI1	-779	KBTBD12	-475	ZNF667	-171	LRAT	134
HOXA3	-1081	OR51E1	-778	FLJ45983	-474	HOXC11	-170	PLAC2	135
GPR45	-1080	LGALS12	-777	UGT2A1	-473	PRR11	-169	IRF7	136
MELK	-1079	MCM10	-776	TNIK	-472	RBM20	-168	HERC6	137

KRTAP5-8	-1078	LOC440356	-775	C9orf140	-471	CALY	-167	DPP6	138
KIF12	-1077	CCNE2	-774	DRD1	-470	PERP	-166	KCNT1	139
ARC	-1076	PDE3A	-773	CLIP3	-469	NPM2	-165	IFI44	140
ALPK3	-1075	LIPH	-772	BLM	-468	ADAMTS6	-164	MX1	141
SLC47A1	-1074	FAM9C	-771	NUF2	-467	ENPP1	-163	IFI6	142
ARAP3	-1073	OXCT2	-770	FSHR	-466	RNFT2	-162	FAM167A	143
KALRN	-1072	NWD1	-769	TEX9	-465	UGT1A6	-161	IFI44L	144
CENPK	-1071	C12orf39	-768	TRHDE	-464	SPATA12	-160	C15orf48	145
TDRD12	-1070	TAS2R46	-767	MIA	-463	HTR2A	-159	IGSF1	146
MMP2	-1069	C7orf57	-766	DMP1	-462	ODZ3	-158	CLGN	147
PRC1	-1068	ABCB5	-765	MMP13	-461	PCDH20	-157	ACSM1	148
KDELC1	-1067	THSD1	-764	ACTG2	-460	MSH4	-156	RSAD2	149
KCNN4	-1066	CTXN2	-763	ZNF699	-459	CPNE7	-155	REG1B	150
GALNTL6	-1065	CENPW	-762	PSD2	-458	SERPINE1	-154	RPRM	151
S100A5	-1064	CDC45	-761	ST6GALNAC3	-457	MMP7	-153	TRIM55	152
C2orf65	-1063	IRS1	-760	RACGAP1P	-456	RASL11B	-152	PEX5L	153
CCDC113	-1062	AKAP14	-759	ALX1	-455	DLX1	-151	IL28A	154
PORCN	-1061	PBX4	-758	KHDRBS2	-454	C5orf46	-150	CBLN1	155
NELL2	-1060	NEK2	-757	TBXA2R	-453	SEMA3D	-149	LOC100132354	156
ABCC4	-1059	IGF2BP1	-756	CPT1C	-452	KRT80	-148	C1orf173	157
LRRC10B	-1058	KCNMA1	-755	THBS2	-451	TNNC1	-147	SLC4A4	158
KIAA1462	-1057	GPR3	-754	GPR87	-450	IQUB	-146	UPK1B	159
PM20D1	-1056	PROM1	-753	BUB1	-449	TPSG1	-145	PAH	160
LOC653653	-1055	MGAM	-752	AADAC	-448	FAT3	-144	TJP3	161
C10orf55	-1054	AQP5	-751	MURC	-447	DLL3	-143	PPARGC1A	162
PRAME	-1053	TPPP3	-750	DYNC1I1	-446	DLX5	-142	SLC26A9	163
MTMR9L	-1052	LGALS7B	-749	GJC1	-445	FBN2	-141	ATP6V1B1	164
GJB3	-1051	HOXA1	-748	CYP27B1	-444	MAST1	-140	LPHN3	165
SLC2A10	-1050	ARHGEF3	-747	PTTG3P	-443	ANKRD30B	-139	ITPKA	166
FOSL1	-1049	ZNF389	-746	IGSF10	-442	SPRR2D	-138	CXorf49B	167
C6orf227	-1048	LOC285629	-745	C6orf168	-441	DPEP1	-137	KIR2DL3	168
PVRL4	-1047	GEM	-744	KIF23	-440	CACNA2D1	-136	IFIT2	169
HRNBP3	-1046	SLC7A5	-743	DNM1	-439	TTLL7	-135	KCNA1	170
CDK15	-1045	CENPF	-742	DKKL1	-438	TRIM50	-134	FLJ42875	171
C16orf45	-1044	ABI3BP	-741	CNGB1	-437	VSTM2L	-133	SAA1	172
ZNF883	-1043	PRR16	-740	ZIC1	-436	FGF5	-132	ANXA9	173
WNT3A	-1042	REEP6	-739	PODXL	-435	SLC12A8	-131	MT1DP	174
KIAA1211	-1041	CCNE1	-738	KRT17	-434	CENPI	-130	TP53AIP1	175
FRMD7	-1040	KIAA1486	-737	MYEF2	-433	COL11A1	-129	TRIM31	176
CXCL13	-1039	HSD17B2	-736	LOC100216001	-432	CD24	-128	SSTR1	177
SOX5	-1038	SSC5D	-735	GUCY1B2	-431	PCYT1B	-127	LIPF	178
ZNF284	-1037	ADORA2B	-734	ANKRD1	-430	FILIP1L	-126	SERPINB7	179
MAP3K13	-1036	SLC22A1	-733	EXO1	-429	ZBTB8B	-125	KCNB2	180
NRADDP	-1035	DEPDC1B	-732	FAM24B	-428	IGFL2	-124	SPACA4	181
THY1	-1034	C7orf10	-731	TNFRSF11B	-427	TNC	-123	RTP4	182
MXRA5	-1033	C2orf66	-730	RASD2	-426	ARNTL2	-122	RARRES3	183
LEMD1	-1032	SEC14L2	-729	PTPRR	-425	KIAA1199	-121	POU3F3	184
UGCG	-1031	RHOBTB3	-728	KIF4A	-424	UTS2	-120	NOX1	185
TMSB15B	-1030	KIF15	-727	SNORD116-4	-423	VIT	-119	ESR2	186
MAPK10	-1029	SPC25	-726	SLC6A4	-422	MLLT11	-118	C12orf26	187
HIST1H4A	-1028	ITIH5	-725	C10orf140	-421	HTR6	-117	RICH2	188
LY6D	-1027	C3orf36	-724	PTHLH	-420	FAM163A	-116	PKHD1L1	189
RUNX2	-1026	CEP55	-723	C2orf89	-419	SLC5A12	-115	IFIT1	190
RAD51AP2	-1025	CENPE	-722	ART4	-418	PTPN13	-114	EPHB6	191
KLHDC8A	-1024	NCRNA00175	-721	RBM24	-417	SPRR2E	-113	C6orf208	192
HOXA6	-1023	TMEM92	-720	ST6GALNAC1	-416	LRRC49	-112	CACHD1	193
C5orf23	-1022	SEMA3A	-719	SERPINB13	-415	GPC2	-111	DAAM1	194
LOC84740	-1021	TSPAN5	-718	SPSB4	-414	SLC16A10	-110	GPBAR1	195
TNFAIP6	-1020	RRAD	-717	ZFP28	-413	SBSN	-109	HRASLS5	196
LOC144486	-1019	CSDC2	-716	MYOZ2	-412	SYTL5	-108	C10orf105	197
JAM3	-1018	DAB1	-715	CDH8	-411	UGT1A10	-107	AMN	198
AFP	-1017	TUBA4B	-714	MYLK	-410	SV2A	-106	IFIT3	199
PCDH7	-1016	KIRREL3	-713	SLC5A4	-409	EDIL3	-105	MAT1A	200
HES7	-1015	MYBL2	-712	HGF	-408	SHANK1	-104	LOC286002	201
DCBLD2	-1014	KIAA0895	-711	TMEM200A	-407	SYT10	-103	BBOX1	202
CBLC	-1013	ZYG11A	-710	RNF43	-406	GPR63	-102	CHRDL2	203
C13orf30	-1012	CCDC54	-709	LRRC15	-405	ZNF365	-101	CPO	204
PSRC1	-1011	CSPG4	-708	STMN2	-404	ADAMTS14	-100	STAC2	205
MYOD1	-1010	SPRR2G	-707	SPRR2B	-403	KRT7	-99	ISG15	206
ASAM	-1009	CHRNA1	-706	SALL1	-402	SPRR2F	-98	NNAT	207
NRIP3	-1008	AFF2	-705	ICAM5	-401	GLYATL2	-97	KRTDAP	208
TNS4	-1007	SGOL1	-704	ACTN3	-400	UCN2	-96	TLR3	209
CHST5	-1006	CPS1	-703	LIX1	-399	SFTA1P	-95	CKB	210
CYB5R2	-1005	SKA3	-702	C3orf32	-398	CHST6	-94	RAB40A	211
TK1	-1004	SPESP1	-701	SULT4A1	-397	STRA6	-93	CCL11	212
CDKN3	-1003	PRND	-700	BPIL2	-396	GPC6	-92	KCNK5	213
TMEM163	-1002	MYLK2	-699	FSIP1	-395	IVL	-91	INMT	214
MGC16121	-1001	RSPH9	-698	C9orf4	-394	OXTR	-90	RAB40AL	215
FGF10	-1000	RASAL2	-697	HAS2AS	-393	ARTN	-89	FCER2	216
IL17D	-999	OIT3	-696	STXBP5L	-392	PRR18	-88	C4BPA	217
XRCC6BP1	-998	CNIH2	-695	TTBK1	-391	GALNT5	-87	GLIPR1L2	218
FAM135B	-997	C6orf132	-694	PAX6	-390	LRRIQ1	-86	UNC93A	219
ADAM19	-996	LOC90246	-693	LAYN	-389	IGFN1	-85	ZFY	220
PCDHA4	-995	NEFM	-692	GUCA1A	-388	PPFIA4	-84	PIK3C2B	221
LOC400804	-994	CCDC33	-691	C19orf45	-387	MFAP5	-83	CREG2	222
IL1RL2	-993	C21orf62	-690	CCDC151	-386	VNN1	-82	SOX6	223
C9orf47	-992	UPK1A	-689	UNC13A	-385	BRSK1	-81	LINGO4	224
LRP8	-991	ATRNL1	-688	FADS2	-384	CA12	-80	PDZD3	225
CPA1	-990	TMEM132A	-687	DEPDC7	-383	CD109	-79	SLC6A3	226

RND1	-989	GALNT8	-686	DMD	-382	IBSP	-78	ADRA1B	227
ETV1	-988	AQP6	-685	HSF2BP	-381	MYOM3	-77	SLC19A3	228
PSD3	-987	HPCAL4	-684	COL24A1	-380	CDH10	-76	FCN1	229
FAM72A	-986	FAM26E	-683	AQP10	-379	SHC3	-75	HIST1H1C	230
LOC286467	-985	ZNF677	-682	ZNF135	-378	KIAA1751	-74	RAX2	231
TRPA1	-984	TMEM158	-681	TSPAN11	-377	DPF1	-73	TST	232
OBP2A	-983	OCLN	-680	DES	-376	NPY	-72	CSAG3	233
ZNF423	-982	TMEFF2	-679	RNF128	-375	ALPK2	-71	SLC6A14	234
TXNDC2	-981	SERPINB5	-678	MESTIT1	-374	PALM3	-70	SUSD4	235
PNPLA3	-980	FANCB	-677	KIF3C	-373	RGS4	-69	TMEM114	236
KIAA1549	-979	COL10A1	-676	C10orf107	-372	TDRD6	-68	C14orf176	237
CCR8	-978	SNAI1	-675	CCDC150	-371	LRCH2	-67	KLHL31	238
SLC27A5	-977	ENTHD1	-674	ACHE	-370	SPOCD1	-66	CYP4F11	239
MAGEC3	-976	ZNF221	-673	EYA4	-369	ITGB6	-65	HS6ST3	240
FAM157A	-975	LPO	-672	PDZD7	-368	GAL3ST3	-64	OAS1	241
RUNDC2C	-974	TBX20	-671	KIF4B	-367	E2F8	-63	STAP1	242
KRT16	-973	USP54	-670	HOTAIR	-366	NPPB	-62	PKD1L2	243
EXTL1	-972	TMEM200C	-669	HDGFRP3	-365	INHBA	-61	FAM134B	244
COL2A1	-971	TM4SF20	-668	DAZL	-364	C6orf141	-60	CYP11A1	245
PLK1	-970	INPP4B	-667	SRRM4	-363	MGC45800	-59	FBXO39	246
PDLIM7	-969	HAND2	-666	GCKR	-362	IL13RA2	-58	TNFSF13B	247
MOSPD1	-968	GATSL2	-665	SCN2B	-361	XIRP1	-57	DGCR5	248
HTATIP2	-967	COL3A1	-664	OR51E2	-360	SUGT1P1	-56	SAA2	249
GPR152	-966	SCUBE1	-663	SLC16A11	-359	BEST3	-55	IL18BP	250
ADD2	-965	KCTD14	-662	ADAMTSL5	-358	RSPO2	-54	KCNA6	251
MFHAS1	-964	CRYM	-661	TFF3	-357	SLC38A4	-53	LAMB3	252
SYNGR3	-963	GRID2	-660	ANKRD43	-356	TRIM46	-52	SAA4	253
DTL	-962	CRABP1	-659	CDCA2	-355	EPYC	-51	ANKK1	254
CNN2	-961	TRDN	-658	VSX1	-354	F5	-50	MUC16	255
CDK1	-960	C12orf63	-657	SLC25A27	-353	LOC222699	-49	RGS22	256
NOX4	-959	OLFML2B	-656	PIF1	-352	DACT2	-48	USP18	257
DCHS2	-958	C11orf82	-655	C21orf125	-351	MOV10L1	-47	GRAMD1C	258
C21orf84	-957	AQP2	-654	TYRP1	-350	UGT1A1	-46	LOC400696	259
AK5	-956	LOC151174	-653	SLC45A1	-349	SYT5	-45	ZBTB16	260
SNTG1	-955	CXXC4	-652	ABCG4	-348	TSPAN2	-44	C13orf36	261
SLC17A9	-954	ORC1L	-651	TEX15	-347	ODZ2	-43	BCO2	262
PNPLA1	-953	DEPDC1	-650	ENC1	-346	NCRNA00189	-42	IL29	263
GBA3	-952	PADI3	-649	DSG3	-345	ADAMTS5	-41	C14orf68	264
TRH	-951	CYP2B7P1	-648	TMEM90B	-344	PHEX	-40	PDZK1IP1	265
SLC7A5P1	-950	KRTAP5-2	-647	STAC	-343	IGFL3	-39	LOC644165	266
LOC442421	-949	GLB1L3	-646	ROS1	-342	UGT1A9	-38	C17orf102	267
AKR1B15	-948	CCDC148	-645	CILP	-341	LOC554202	-37	LOC146336	268
SMPX	-947	TNIP3	-644	KCNQ5	-340	CLDN1	-36	CSAG1	269
PRAP1	-946	NPY6R	-643	CCL28	-339	SLC35D3	-35	GHRHR	270
CNTNAP1	-945	C11orf20	-642	PKP3	-338	GPR176	-34	LOC257358	271
ADAM22	-944	CDC20	-641	HMSD	-337	CDH2	-33	LMX1B	272
ZNF625	-943	KIAA1614	-640	MGC4473	-336	FAM40B	-32	SCARA3	273
ULBP3	-942	INPP5J	-639	ANXA13	-335	CAMK2A	-31	ADCY3	274
SOX2	-941	AKR1E2	-638	DAND5	-334	MUC12	-30	SELENBP1	275
GTF2A1L	-940	TNNT1	-637	KRT13	-333	KLHL38	-29	TNNI3K	276
ZNF833	-939	PGBD1	-636	SP9	-332	ISL2	-28	FHDC1	277
RGS17	-938	CUZD1	-635	LOC157381	-331	CCBE1	-27	IFI27	278
FAM179A	-937	TGM4	-634	RANBP3L	-330	HSPB3	-26	TCERG1L	279
SLC7A11	-936	L1CAM	-633	LARP6	-329	IGF2BP2	-25	SMAD5OS	280
S100A3	-935	LOC100133893	-632	FN1	-328	APCDD1L	-24	CRHBP	281
GLI1	-934	FOXC2	-631	TMC3	-327	CLDN16	-23	SGPP2	282
C19orf71	-933	SLC45A2	-630	IL12B	-326	SULT1B1	-22	TGM3	283
HKDC1	-932	KRT75	-629	BCAS1	-325	C12orf68	-21	SLC28A3	284
ZNF222	-931	PAX3	-628	ENOX1	-324	CYP39A1	-20	ZNF676	285
SEC16B	-930	NOS3	-627	RBMS3	-323	FRMD5	-19	CCDC135	286
AMBP	-929	GLDN	-626	CYP2C8	-322	SLC22A3	-18	C11orf67	287
F2R	-928	C1orf92	-625	TIAM2	-321	SBK2	-17	MREG	288
MEST	-927	TREM1	-624	LIN7A	-320	SHOX2	-16	HIST1H2AC	289
FOXS1	-926	FAM54A	-623	DIRAS2	-319	LHX9	-15	FAM150A	290
PTPN20B	-925	SLC22A2	-622	GIP	-318	KCNN2	-14	RBM46	291
HUS1B	-924	ME1	-621	WDR66	-317	INA	-13	CYP4B1	292
GRIK1	-923	ZNF439	-620	ARSI	-316	IGFBP1	-12	FAM3B	293
AXL	-922	CKAP2L	-619	UNC5C	-315	ADAMTS16	-11	KIF26A	294
H2BFXP	-921	IRX4	-618	DACT1	-314	SEC14L4	-10	LAG3	295
OTX1	-920	CALB1	-617	CLDN14	-313	SULT1E1	-9	OVOL2	296
DNAH2	-919	ARL10	-616	ISL1	-312	DKFZp434J022	-8	PHLPP2	297
CCDC158	-918	MYO1B	-615	KAAG1	-311	RASSF9	-7	SCEL	298
ADAM12	-917	C6orf124	-614	TLL1	-310	TUBB2B	-6	CYP3A43	299
WDR49	-916	TPRG1	-613	SH3GL3	-309	KRT14	-5	HERC5	300
FBXO32	-915	NCAPH	-612	B4GALNT1	-308	NCRNA00162	-4	KGFLP2	301
BCAT1	-914	TP53TG3B	-611	HOXA11AS	-307	DKK1	-3	RASGRP4	302
IGSF9B	-913	EDNRA	-610	SGEF	-306	RAB3B	-2	NFKBIZ	303
C3orf55	-912	SOAT2	-609	SEMA3E	-305	KCNS1	-1	KCNA5	304
ZSWIM4	-911	PPFIA2	-608	PRR5L	-304	ADH1A	1	ALOX12B	305
CYP2S1	-910	NBPF16	-607	LTBP1	-303	MRAP	2	FLJ35024	306
ARL3	-909	NGFR	-606	SKA1	-302	CHST4	3	ASPA	307
SCT	-908	ZNF471	-605	GLP2R	-301	FAM83E	4	GDPD5	308
RPA4	-907	TMEM233	-604	TGFB1I1	-300	C14orf64	5	SLC14A1	309
RDM1	-906	TFR2	-603	PTPLB	-299	VIPR2	6	CARNS1	310
GSDMC	-905	ZNF385D	-602	KLHL4	-298	ACE2	7	HIST1H1T	311
FOXP2	-904	TNFSF11	-601	LCA5	-297	DSCAML1	8	FAM164C	312
SLC30A10	-903	ISLR2	-600	PKD1L1	-296	PRIMA1	9	GRIA1	313
NKAIN2	-902	HHIP	-599	ANLN	-295	UGT3A1	10	ZBP1	314
LCTL	-901	CSRP3	-598	IPMK	-294	GAS2	11	AJAP1	315

DCDC1	-900	PRSS3	-597	MYBPC2	-293	PRR15L	12	HIST1H2BC	316
KCNH4	-899	C3orf67	-596	ZNF608	-292	OLIG1	13	PLB1	317
LOC55908	-898	ERCC6L	-595	TM6SF2	-291	LIPM	14	CES4	318
TMEM231	-897	DENND2A	-594	LOC730668	-290	DMRTC1B	15	RSPO4	319
IFNE	-896	KIF20A	-593	FAM64A	-289	ADH1B	16	C10orf95	320
WDR62	-895	ZIC2	-592	BACH2	-288	ADH6	17	GHR	321
MIR155HG	-894	HES2	-591	FRG2C	-287	RUFY4	18	PLEKHH2	322
AVPR1A	-893	XK	-590	FIBCD1	-286	SLC14A2	19	ACVR1C	323
TSNAX-DISC1	-892	EPHA3	-589	SLITRK6	-285	PKHD1	20	GPM6A	324
NFE2L3	-891	CDH6	-588	INHBE	-284	PKD1L3	21	PCDH11Y	325
TRIP13	-890	CCIN	-587	MEIS3	-283	ISM2	22	PCDHGB2	326
NLRP14	-889	KIAA1324	-586	F2RL3	-282	REG1A	23	CYS1	327
KRT71	-888	LBX1	-585	KLC3	-281	CDKN2A	24	CD209	328
ITGA10	-887	PTPRN	-584	C19orf26	-280	TGM5	25	CXCL6	329
FAM131C	-886	RIBC2	-583	MCHR1	-279	LOC154822	26	B3GNT7	330
LOC121838	-885	PDE4C	-582	C10orf67	-278	TUBAL3	27	POU2F3	331
C17orf100	-884	LYPD1	-581	MGAT4C	-277	RDH16	28	FSTL4	332
SATB2	-883	HERC2P4	-580	NMNAT2	-276	NRG3	29	LOC100128788	333
GRIA2	-882	CHRNA9	-579	LCNL1	-275	LOC100133469	30	RSPO1	334
GOLGA8C	-881	TRPM3	-578	C6orf186	-274	CYP8B1	31	ALPI	335
SLC17A7	-880	SNX32	-577	GDNF	-273	CD300LD	32	EPB42	336
NAV2	-879	KCNK12	-576	BEND6	-272	CCL7	33	NT5C3	337
FLJ30679	-878	EMR4P	-575	MMP23A	-271	RBP4	34	HLF	338
ELAVL3	-877	SPRR2A	-574	BTNL8	-270	SORBS2	35	PAK7	339
CCDC68	-876	CDCA7	-573	TRIM54	-269	FGF7	36	OASL	340
ATP6V0A4	-875	FHOD3	-572	LOXL2	-268	KCNH5	37	S100A12	341
SLC12A5	-874	KCNAB3	-571	C8orf84	-267	PLA2G1B	38	IFIH1	342
ARMC3	-873	DNAH3	-570	DLX3	-266	BTBD16	39	C9	343
PMAIP1	-872	ZCCHC18	-569	CCNA1	-265	CXCL1	40	HIST1H2BK	344
MYOZ3	-871	LEPREL2	-568	IL5RA	-264	HP	41	CA4	345
UGT1A7	-870	LDLR	-567	ATG9B	-263	OR4N2	42	CYB5A	346
IL1A	-869	LOC441204	-566	SNAP25	-262	HPR	43	KIR3DL1	347
KRT9	-868	KRT79	-565	SYNPO2L	-261	LCN2	44	GFPT2	348
VCAN	-867	FMN2	-564	GPR156	-260	AGXT2L1	45	KANK4	349
NOS2	-866	ALOX15B	-563	MAP6	-259	CRB1	46	PLLP	350
ARX	-865	XRCC2	-562	GSDMA	-258	PCDH11X	47	SIGLEC16	351
ULBP1	-864	DST	-561	HMGA2	-257	RAET1G	48	CYP4F12	352
FOXD3	-863	NKX2-5	-560	GNB3	-256	BARX2	49	REC8	353
TNFSF4	-862	SLITRK1	-559	COL5A2	-255	NFE2	50	ALG1L	354
TMEM90A	-861	TCEAL7	-558	KERA	-254	C17orf77	51	NXNL2	355
TCAM1P	-860	S1PR3	-557	SLITRK3	-253	TAT	52	PHYHIP	356
SGCE	-859	OXGR1	-556	ARHGAP6	-252	DNAH9	53	RIMKLA	357
		NCRNA00181	-555	NALCN	-251	CXCR2P1	54	PLA2G2A	358
								IL1RN	359
