# Supplementary material for: Define the Two Molecular Subtypes of Epithelioid Malignant Pleural Mesothelioma
Source: Cells. 2022 Sep 19;11(18):2924. doi: 10.3390/cells11182924 (PMC9497219; doi:10.3390/cells11182924)
Supplement: Supplementary file 1 [file cells-11-02924-s001.zip › cells-1854477-Table S3.pdf]

Table S3. Biological process enriched in each subtypes of Mesothelioma. Enriched GO BP terms by genes over-expressed in Subtype I.

Category	Term	Count	%	PValue	Genes	List Total	Pop Hits	Pop Total	Fold Enrich	Bonferroni	Benjamini	FDR
GOTERM_BP_DIRECT	GO:1904224~negative regulation of glucurono	5	2.73224	4.87E-07	UGT1A10, UGT1A1, UG	157	8	16792	66.84713	4.28E-04	1.91E-04	1.90E-04
GOTERM_BP_DIRECT	GO:2001030~negative regulation of cellular gl u	5	2.73224	4.87E-07	UGT1A10, UGT1A1, UG	157	8	16792	66.84713	4.28E-04	1.91E-04	1.90E-04
GOTERM_BP_DIRECT	GO:0045922~negative regulation of fatty acid me	5	2.73224	8.71E-07	UGT1A10, UGT1A1, UG	157	9	16792	59.41967	7.64E-04	1.91E-04	1.90E-04
GOTERM_BP_DIRECT	GO:0052697~xenobiotic glucuronidation	5	2.73224	8.71E-07	UGT1A10, UGT1A1, U	157	9	16792	59.41967	7.64E-04	1.91E-04	1.90E-04
GOTERM_BP_DIRECT	GO:0052695~cellular glucuronidation	5	2.73224	1.20E-05	UGT1A10, UGT1A1, U	157	16	16792	33.42357	0.010444	0.0021	0.002085
GOTERM_BP_DIRECT	GO:0051552~flavone metabolic process	4	2.185792	1.54E-05	UGT1A10, UGT1A1, U	157	6	16792	71.30361	0.01344	0.002255	0.00224
GOTERM_BP_DIRECT	GO:0052696~flavonoid glucuronidation	5	2.73224	4.60E-05	UGT1A10, UGT1A1, U	157	22	16792	24.30805	0.039599	0.005772	0.005732
GOTERM_BP_DIRECT	GO:0030198~extracellular matrix organizatio	10	5.464481	9.12E-05	FBN2, VIT, MFAP5, A	157	196	16792	5.456909	0.076946	0.010008	0.00994
GOTERM_BP_DIRECT	GO:0007268~chemical synaptic transmission	9	4.918033	0.001833	SYT5, HTR6, GPR176	157	240	16792	4.010828	0.800221	0.178785	0.177564
GOTERM_BP_DIRECT	GO:0022617~extracellular matrix disassembly	5	2.73224	0.005476	FBN2, ACAN, BCAN,	157	76	16792	7.03654	0.991944	0.480814	0.477529
GOTERM_BP_DIRECT	GO:0042573~retinoic acid metabolic process	3	1.639344	0.007252	UGT1A1, UGT1A9, U	157	14	16792	22.91902	0.998323	0.521622	0.518058
GOTERM_BP_DIRECT	GO:0008202~steroid metabolic process	4	2.185792	0.007395	SULT1B1, UGT1A1, S	157	43	16792	9.949341	0.998522	0.521622	0.518058
GOTERM_BP_DIRECT	GO:0002063~chondrocyte development	3	1.639344	0.008317	ACAN, COL11A1, SH	157	15	16792	21.39108	0.999347	0.521622	0.518058
GOTERM_BP_DIRECT	GO:0050427~3'-phosphoadenosine 5'-phospl	3	1.639344	0.008317	SULT1B1, SULT1E1,	157	15	16792	21.39108	0.999347	0.521622	0.518058
GOTERM_BP_DIRECT	GO:0001501~skeletal system development	6	3.278689	0.009088	ACAN, BCAN, DLX5,	157	137	16792	4.684179	0.99967	0.531933	0.528298
GOTERM_BP_DIRECT	GO:0031424~keratinization	4	2.185792	0.010021	SPRR2E, SPRR2F, IVL	157	48	16792	8.912951	0.999856	0.549902	0.546144
GOTERM_BP_DIRECT	GO:0018149~peptide cross-linking	4	2.185792	0.011204	SPRR2E, SPRR2F, IVL	157	50	16792	8.556433	0.999949	0.578664	0.574709
GOTERM_BP_DIRECT	GO:0009813~flavonoid biosynthetic process	3	1.639344	0.014601	UGT1A10, UGT1A1, U	157	20	16792	16.04331	0.999998	0.712203	0.707336
GOTERM_BP_DIRECT	GO:0001649~osteoblast differentiation	5	2.73224	0.01609	IBSP, DLX5, SHOX2,	157	104	16792	5.142087	0.999999	0.74353	0.738449
GOTERM_BP_DIRECT	GO:0045939~negative regulation of steroid m	2	1.092896	0.018495	UGT1A1, UGT1A8	157	2	16792	106.9554	1	0.811909	0.806361
GOTERM_BP_DIRECT	GO:0007586~digestion	4	2.185792	0.020807	CYP39A1, UGT1A1, U	157	63	16792	6.79082	1	0.869922	0.863977
GOTERM_BP_DIRECT	GO:0007507~heart development	6	3.278689	0.028139	ACAN, OXTR, SHOX2	157	183	16792	3.506735	1	1	0.994299
GOTERM_BP_DIRECT	GO:0030203~glycosaminoglycan metabolic pr	3	1.639344	0.029551	BCAN, GPC2, GPC6	157	29	16792	11.06435	1	1	0.994299
GOTERM_BP_DIRECT	GO:0060325~face morphogenesis	3	1.639344	0.031472	DLX5, DKK1, STRA6	157	30	16792	10.69554	1	1	0.994299
GOTERM_BP_DIRECT	GO:0030216~keratinocyte differentiation	4	2.185792	0.033779	SPRR2E, SPRR2F, IVL	157	76	16792	5.629232	1	1	0.994299
GOTERM_BP_DIRECT	GO:0006805~xenobiotic metabolic process	4	2.185792	0.036073	SULT1B1, DPEP1, UG	157	78	16792	5.484893	1	1	0.994299
GOTERM_BP_DIRECT	GO:0046959~habituation	2	1.092896	0.036649	DGKI, SHANK1	157	4	16792	53.47771	1	1	0.994299
GOTERM_BP_DIRECT	GO:0030282~bone mineralization	3	1.639344	0.039625	IBSP, RSPO2, PHEX	157	34	16792	9.437242	1	1	0.994299
GOTERM_BP_DIRECT	GO:0017158~regulation of calcium ion-deper	3	1.639344	0.041776	SYT5, SYT10, SYTL5	157	35	16792	9.167607	1	1	0.994299
GOTERM_BP_DIRECT	GO:0006508~proteolysis	10	5.464481	0.043973	ADAMTS16, ACAN, A	157	500	16792	2.139108	1	1	0.994299
GOTERM_BP_DIRECT	GO:0008544~epidermis development	4	2.185792	0.044713	SPRR2E, SPRR2F, KR	157	85	16792	5.033196	1	1	0.994299
GOTERM_BP_DIRECT	GO:0046883~regulation of hormone secretio	2	1.092896	0.045601	SCG5, HTR2A	157	5	16792	42.78217	1	1	0.994299
GOTERM_BP_DIRECT	GO:0034332~adherens junction organization	3	1.639344	0.046205	CDH2, CDH10, CDH1	157	37	16792	8.672061	1	1	0.994299
GOTERM_BP_DIRECT	GO:0001942~hair follicle development	3	1.639344	0.048482	CD109, INHBA, DKK1	157	38	16792	8.443848	1	1	0.994299
GOTERM_BP_DIRECT	GO:0048791~calcium ion-regulated exocytosi	3	1.639344	0.050798	SYT5, SYT10, SYTL5	157	39	16792	8.22734	1	1	0.994299
GOTERM_BP_DIRECT	GO:0030199~collagen fibril organization	3	1.639344	0.050798	ACAN, ADAMTS14, C	157	39	16792	8.22734	1	1	0.994299
GOTERM_BP_DIRECT	GO:0007399~nervous system development	7	3.825137	0.05165	TTLL7, FGF5, DLX5, D	157	287	16792	2.608669	1	1	0.994299
GOTERM_BP_DIRECT	GO:0030326~embryonic limb morphogenesis	3	1.639344	0.053154	FBN2, DLX5, DKK1	157	40	16792	8.021656	1	1	0.994299
GOTERM_BP_DIRECT	GO:0006024~glycosaminoglycan biosynthetic	3	1.639344	0.057979	GALNT5, GPC2, GPC	157	42	16792	7.639672	1	1	0.994299
GOTERM_BP_DIRECT	GO:0001541~ovarian follicle development	3	1.639344	0.057979	PCYT1B, MSH4, INHE	157	42	16792	7.639672	1	1	0.994299
GOTERM_BP_DIRECT	GO:0007156~homophilic cell adhesion via pla	5	2.73224	0.059887	CDH2, CDH10, PCDH	157	158	16792	3.384665	1	1	0.994299
GOTERM_BP_DIRECT	GO:0007155~cell adhesion	9	4.918033	0.064827	ACAN, BCAN, IBSP, C	157	459	16792	2.097165	1	1	0.994299
GOTERM_BP_DIRECT	GO:0008152~metabolic process	5	2.73224	0.071606	UGT1A10, UGT1A1, U	157	168	16792	3.183197	1	1	0.994299
GOTERM_BP_DIRECT	GO:0007269~neurotransmitter secretion	3	1.639344	0.081394	BRSK1, PPFIA4, DGKI	157	51	16792	6.291495	1	1	0.994299
GOTERM_BP_DIRECT	GO:0032098~regulation of appetite	2	1.092896	0.089135	SLC22A3, NPY	157	10	16792	21.39108	1	1	0.994299
GOTERM_BP_DIRECT	GO:0051923~sulfation	2	1.092896	0.097602	SULT1B1, SULT1E1	157	11	16792	19.44644	1	1	0.994299
GOTERM_BP_DIRECT	GO:0008210~estrogen metabolic process	2	1.092896	0.097602	UGT1A1, SULT1E1	157	11	16792	19.44644	1	1	0.994299
GOTERM_BP_DIRECT	GO:0060272~embryonic skeletal joint morph	2	1.092896	0.097602	SHOX2, HOXC11	157	11	16792	19.44644	1	1	0.994299
GOTERM_BP_DIRECT	GO:0030431~sleep	2	1.092896	0.097602	OXTR, HTR2A	157	11	16792	19.44644	1	1	0.994299
GOTERM_BP_DIRECT	GO:0061045~negative regulation of wound h	2	1.092896	0.097602	CD109, SERPINE1	157	11	16792	19.44644	1	1	0.994299

Enriched GO BP terms by genes over-expressed in Subtype II Mesothelioma.

Category	Term	Count	%	PValue	Genes	List Total	Pop Hits	Pop Total	Fold Enric	Bonferron	Benjamini	FDR
GOTERM_BP_DIRECT	GO:0009615~response to virus	9	5	1.07E-05	RSAD2, MX1, IRF7, L	162	110	16792	8.480808	0.009041	0.009082	0.009082
GOTERM_BP_DIRECT	GO:0060337~type I interferon signaling pathv	7	3.888889	3.35E-05	RSAD2, MX1, IRF7, I	162	64	16792	11.33719	0.027956	0.014177	0.014177
GOTERM_BP_DIRECT	GO:0051607~defense response to virus	8	4.444444	0.001071	RSAD2, DMBT1, MX1	162	165	16792	5.025664	0.595949	0.301909	0.301909
GOTERM_BP_DIRECT	GO:0034765~regulation of ion transmembrar	6	3.333333	0.004316	KCNH5, KCND3, KCN	162	111	16792	5.602936	0.97424	0.912756	0.912756
GOTERM_BP_DIRECT	GO:0006069~ethanol oxidation	3	1.666667	0.005661	ADH1B, ADH1A, ADH	162	12	16792	25.91358	0.991794	0.957854	0.957854
GOTERM_BP_DIRECT	GO:0006813~potassium ion transport	5	2.777778	0.007978	SLC24A3, KCND3, KC	162	82	16792	6.320385	0.99886	1	1
GOTERM_BP_DIRECT	GO:0016042~lipid catabolic process	5	2.777778	0.009035	LIPF, RARRES3, PLA2	162	85	16792	6.097313	0.999537	1	1
GOTERM_BP_DIRECT	GO:0072236~metanephric loop of Henle deve	2	1.111111	0.019084	WNT7B, POU3F3	162	2	16792	103.6543	1	1	1
GOTERM_BP_DIRECT	GO:0006898~receptor-mediated endocytosis	6	3.333333	0.033625	AMN, DMBT1, ALB,	162	186	16792	3.343688	1	1	1
GOTERM_BP_DIRECT	GO:0042391~regulation of membrane potent	4	2.222222	0.035368	KCNH5, KCNA1, SLC	162	75	16792	5.52823	1	1	1
GOTERM_BP_DIRECT	GO:0007267~cell-cell signaling	7	3.888889	0.035678	CRB1, VIPR2, CCL7,	162	254	16792	2.856615	1	1	1
GOTERM_BP_DIRECT	GO:0014066~regulation of phosphatidylinosi	4	2.222222	0.039037	BTC, FGF7, NRG4, PI	162	78	16792	5.315606	1	1	1
GOTERM_BP_DIRECT	GO:0042594~response to starvation	3	1.666667	0.044221	SSTR1, PPARGC1A, D	162	35	16792	8.884656	1	1	1
GOTERM_BP_DIRECT	GO:0051902~negative regulation of mitochor	2	1.111111	0.047034	HSH2D, IFI6	162	5	16792	41.46173	1	1	1
GOTERM_BP_DIRECT	GO:0038170~somatostatin signaling pathway	2	1.111111	0.047034	SSTR1, SSTR5	162	5	16792	41.46173	1	1	1
GOTERM_BP_DIRECT	GO:0003081~regulation of systemic arterial b	2	1.111111	0.056174	ACE2, NOX1	162	6	16792	34.55144	1	1	1
GOTERM_BP_DIRECT	GO:0001895~retina homeostasis	3	1.666667	0.056211	ARMS2, ALB, AIPL1	162	40	16792	7.774074	1	1	1
GOTERM_BP_DIRECT	GO:0007190~activation of adenylate cyclase	3	1.666667	0.056211	VIPR2, CRHR1, DRD5	162	40	16792	7.774074	1	1	1
GOTERM_BP_DIRECT	GO:0045071~negative regulation of viral gen	3	1.666667	0.056211	RSAD2, MX1, IFIT1	162	40	16792	7.774074	1	1	1
GOTERM_BP_DIRECT	GO:0055114~oxidation-reduction process	11	6.111111	0.059086	LIPF, C15ORF48, ALD	162	592	16792	1.926009	1	1	1
GOTERM_BP_DIRECT	GO:0006874~cellular calcium ion homeostasi	4	2.222222	0.060117	PKHD1, SLC24A3, CC	162	93	16792	4.45825	1	1	1
GOTERM_BP_DIRECT	GO:0046854~phosphatidylinositol phosphory	4	2.222222	0.061679	BTC, FGF7, NRG4, PI	162	94	16792	4.410822	1	1	1
GOTERM_BP_DIRECT	GO:0008202~steroid metabolic process	3	1.666667	0.063887	HSD17B6, CYP3A4, S	162	43	16792	7.231697	1	1	1
GOTERM_BP_DIRECT	GO:0007187~G-protein coupled receptor sigr	3	1.666667	0.071892	NPY1R, SSTR1, SSTR	162	46	16792	6.760064	1	1	1
GOTERM_BP_DIRECT	GO:0048513~animal organ development	2	1.111111	0.074193	NRG3, NRG4	162	8	16792	25.91358	1	1	1
GOTERM_BP_DIRECT	GO:0008284~positive regulation of cell prolif	9	5	0.080103	PKHD1, BTC, CNTFR,	162	466	16792	2.001907	1	1	1
GOTERM_BP_DIRECT	GO:0035457~cellular response to interferon-	2	1.111111	0.083074	IFIT3, IFIT2	162	9	16792	23.03429	1	1	1
GOTERM_BP_DIRECT	GO:0010579~positive regulation of adenylate	2	1.111111	0.083074	CRHR1, DRD5	162	9	16792	23.03429	1	1	1
GOTERM_BP_DIRECT	GO:0010389~regulation of G2/M transition o	2	1.111111	0.083074	KCNH5, CDKN2A	162	9	16792	23.03429	1	1	1
GOTERM_BP_DIRECT	GO:0010838~positive regulation of keratinoc	2	1.111111	0.083074	TGM1, FGF7	162	9	16792	23.03429	1	1	1
GOTERM_BP_DIRECT	GO:0042359~vitamin D metabolic process	2	1.111111	0.09187	CYP24A1, CYP3A4	162	10	16792	20.73086	1	1	1
