# Supplementary material for: Define the Two Molecular Subtypes of Epithelioid Malignant Pleural Mesothelioma
Source: Cells. 2022 Sep 19;11(18):2924. doi: 10.3390/cells11182924 (PMC9497219; doi:10.3390/cells11182924)
Supplement: Supplementary file 1 [file cells-11-02924-s001.zip › cells-1854477-Table S4.pdf]

Table S4. Biological process enriched in each subtypes of Mesothelioma. Enriched KEGG terms by genes over-expressed in Subtype I.

Category	Term	Count	%	PValue	Genes	List Total	Pop Hits	Pop Total	Fold Enric	Bonferron	Benjamini	FDR
KEGG_PATHWAY	hsa00053:Ascorbate and aldarate metabolis	5	2.73224	5.60E-05	UGT1A10, UGT1A1,	56	27	6879	22.74802	0.005975	0.004495	0.004327
KEGG_PATHWAY	hsa00140:Steroid hormone biosynthesis	6	3.278689	9.01E-05	UGT1A10, UGT1A1,	56	58	6879	12.70751	0.009599	0.004495	0.004327
KEGG_PATHWAY	hsa00040:Pentose and glucuronate interconv	5	2.73224	1.26E-04	UGT1A10, UGT1A1,	56	33	6879	18.61201	0.013394	0.004495	0.004327
KEGG_PATHWAY	hsa00860:Porphyrin and chlorophyll metabol	5	2.73224	3.27E-04	UGT1A10, UGT1A1,	56	42	6879	14.62372	0.034368	0.008742	0.008415
KEGG_PATHWAY	hsa00983:Drug metabolism - other enzymes	5	2.73224	4.65E-04	UGT1A10, UGT1A1,	56	46	6879	13.3521	0.048578	0.009957	0.009585
KEGG_PATHWAY	hsa00830:Retinol metabolism	5	2.73224	0.001629	UGT1A10, UGT1A1,	56	64	6879	9.596819	0.16006	0.029047	0.027961
KEGG_PATHWAY	hsa00982:Drug metabolism - cytochrome P4	5	2.73224	0.002039	UGT1A10, UGT1A1,	56	68	6879	9.0323	0.196193	0.031168	0.030003
KEGG_PATHWAY	hsa00980:Metabolism of xenobiotics by cyto	5	2.73224	0.002781	UGT1A10, UGT1A1,	56	74	6879	8.299952	0.257664	0.037192	0.035802
KEGG_PATHWAY	hsa05204:Chemical carcinogenesis	5	2.73224	0.00369	UGT1A10, UGT1A1,	56	80	6879	7.677455	0.326662	0.043864	0.042224
KEGG_PATHWAY	hsa04512:ECM-receptor interaction	5	2.73224	0.004983	IBSP, SV2A, COL11A	56	87	6879	7.059729	0.41405	0.053319	0.051326
KEGG_PATHWAY	hsa04020:Calcium signaling pathway	5	2.73224	0.05415	HTR6, OXTR, TNNC1,	56	179	6879	3.431265	0.997412	0.526728	0.507038
KEGG_PATHWAY	hsa04024:cAMP signaling pathway	5	2.73224	0.072896	HTR6, OXTR, NPY, C	56	198	6879	3.102002	0.999696	0.649985	0.625687
KEGG_PATHWAY	hsa04510:Focal adhesion	5	2.73224	0.08167	SHC3, IBSP, COL11A	56	206	6879	2.981536	0.99989	0.67221	0.64708
KEGG_PATHWAY	hsa04310:Wnt signaling pathway	4	2.185792	0.097667	MMP7, CAMK2A, FZ	56	138	6879	3.560559	0.999983	0.712584	0.685945
KEGG_PATHWAY	hsa05412:Arrhythmogenic right ventricular c	3	1.639344	0.099895	CDH2, CACNA2D1, I	56	67	6879	5.500267	0.999987	0.712584	0.685945

Enriched KEGG terms by genes over-expressed in Subtype II of Mesothelioma.

Category	Term	Count	%	PValue	Genes	List Total	Pop Hits	Pop Total	Fold Enric	Bonferron	Benjamini	FDR
KEGG_PATHWAY	hsa00830:Retinol metabolism	7	3.888889	7.78E-05	ADH1B, ADH1A, RD	79	64	6879	9.523932	0.009755	0.009803	0.009803
KEGG_PATHWAY	hsa00350:Tyrosine metabolism	4	2.222222	0.007072	ADH1B, TAT, ADH1A	79	35	6879	9.951537	0.591095	0.445553	0.445553
KEGG_PATHWAY	hsa05204:Chemical carcinogenesis	5	2.777778	0.012624	ADH1B, ADH1A, CYP	79	80	6879	5.442247	0.798265	0.530225	0.530225
KEGG_PATHWAY	hsa00982:Drug metabolism - cytochrome P4	4	2.222222	0.041547	ADH1B, ADH1A, CYP	79	68	6879	5.122115	0.995237	1	1
KEGG_PATHWAY	hsa00980:Metabolism of xenobiotics by cyto	4	2.222222	0.051238	ADH1B, ADH1A, CYP	79	74	6879	4.706808	0.998676	1	1
KEGG_PATHWAY	hsa00400:Phenylalanine, tyrosine and trypto	2	1.111111	0.055439	TAT, PAH	79	5	6879	34.83038	0.999243	1	1
KEGG_PATHWAY	hsa00071:Fatty acid degradation	3	1.666667	0.081706	ADH1B, ADH1A, AD	79	42	6879	6.219711	0.999978	1	1
